# Success Counteracting Tobacco Company Interference in Thailand: An Example of FCTC Implementation for Low- and Middle-income Countries

**DOI:** 10.3390/ijerph9041111

**Published:** 2012-03-27

**Authors:** Naowarut Charoenca, Jeremiah Mock, Nipapun Kungskulniti, Sunida Preechawong, Nicholas Kojetin, Stephen L. Hamann

**Affiliations:** 1 Faculty of Public Health, Mahidol University, 272 Rama VI Road, Ratchathewi, Bangkok 10400, Thailand; Email: nao.naowarut@yahoo.com; 2 Center for the Study of Communication-Design, Osaka University, 2-1 Yamada Oka, Osaka 565-0871, Japan; Email: jeremiah.mock@gmail.com; 3 Faculty of Nursing, Chulalongkorn University, 154 Phayathai Road, Pathumwan, Bangkok 10330, Thailand; Email: psunida@gmail.com; 4 Human Development and Family Studies, Indiana University, 107 South Indianan Avenue, Bloomington, IN 47405, USA; Email: nkojetin@umail.iu.edu; 5 Tobacco Control Research and Knowledge Management Center, Mahidol University, 272 Rama VI Road, Ratchathewi, Bangkok 10400, Thailand; Email: slhamann@gmail.com

**Keywords:** tobacco control, transnational tobacco companies, tobacco industry interference, policymaking, Framework Convention on Tobacco Control, Article 5.3, World Health Organization, Thailand

## Abstract

Transnational tobacco companies (TTCs) interfere regularly in policymaking in low- and middle-income countries (LMICs). The WHO Framework Convention for Tobacco Control provides mechanisms and guidance for dealing with TTC interference, but many countries still face ‘how to’ challenges of implementation. For more than two decades, Thailand’s public health community has been developing a system for identifying and counteracting strategies TTCs use to derail, delay and undermine tobacco control policymaking. Consequently, Thailand has already implemented most of the FCTC guidelines for counteracting TTC interference. In this study, our aims are to describe strategies TTCs have used in Thailand to interfere in policymaking, and to examine how the public health community in Thailand has counteracted TTC interference. We analyzed information reported by three groups with a stake in tobacco control policies: Thai tobacco control advocates, TTCs, and international tobacco control experts. To identify TTC viewpoints and strategies, we also extracted information from internal tobacco industry documents. We synthesized these data and identified six core strategies TTCs use to interfere in tobacco control policymaking: (1) doing business with ‘two faces’, (2) seeking to influence people in high places, (3) ‘buying’ advocates in grassroots organizations, (4) putting up a deceptive front, (5) intimidation, and (6) undermining controls on tobacco advertising, promotion and sponsorship. We present three case examples showing where TTCs have employed multiple interference strategies simultaneously, and showing how Thai tobacco control advocates have successfully counteracted those strategies by: (1) conducting vigilant surveillance, (2) excluding tobacco companies from policymaking, (3) restricting tobacco company sales, (4) sustaining pressure, and (5) dedicating resources to the effective enforcement of regulations. Policy implications from this study are that tobacco control advocates in LMICs may be able to develop countermeasures similar to those we identified in Thailand based on FCTC guidelines to limit TTC interference.

## 1. Introduction

Comprehensive tobacco control can contribute to building healthy societies and to reducing chronic diseases [1,2]. One central dimension of comprehensive tobacco control is controlling transnational tobacco company (TTC) interference in policymaking through effective surveillance, regulation and enforcement. Not surprisingly, TTCs do not like to be observed, regulated or compelled to follow laws, and they have a long history of interfering with public health efforts to control their activities [3]. For most TTCs, interference in tobacco control is a core business strategy. In many countries and in many cases, TTCs have been highly effective at employing stealthy strategies and subversive—even illegal—tactics to undermine public health efforts.

Over the past six years, low- and middle-income countries (LMICs) around the world have been mobilizing themselves to establish comprehensive tobacco control under the World Health Organization Framework Convention on Tobacco Control (WHO FCTC) [4]. One of the general obligations under FCTC requires that “in setting and implementing their public health policies with respect to tobacco control, Parties shall act to protect these policies from commercial and other vested interests of the tobacco industry in accordance with national law” [5]. To date, public health communities in many LMICs have faced difficulties preventing TTCs from interfering with public health policymaking and tobacco control programs. One reason is that in LMICs, TTCs have hardly ever been studied [6]. One recent analysis shows that tobacco industry surveillance research has been conducted in less than 10% of LMICs [7].

TTCs have been in the business of interfering with health policymaking in LMICs for about as long as they have been in the business of exporting their products to those countries. The global public health community only recently began to recognize the scope and depth of their interference. As a result, in 2000, during the early period of development of the FCTC, the World Health Assembly (WHA) adopted a resolution to provide guidance to countries about tobacco industry interference. The WHA urged member states to be aware of affiliations between TTCs and their delegations, to assure the integrity of health policy development, and to inform other member states of TTC efforts to impede tobacco control [8].

When a country’s public health community has only a clouded understanding of TTCs’ strategies and tactics, tobacco control efforts are likely to be incomplete, misdirected and ineffective [9]. In many LMICs, public health communities have been engaged in a high-stakes chess match with TTCs, but these public health communities has been playing like a team that can only see half the board. 

Thailand’s public health community has achieved some important successes in efforts to counteract TTCs’ interference. For example, WHO Tobacco-Free Initiative recently recognized Thailand’s efforts in “creating a firewall between the policymakers for tobacco control and the state tobacco monopoly” [10]. In this study, we asked three questions: What strategies have TTCs attempted to use in Thailand to interfere with tobacco control policymaking? What approaches have tobacco control advocates in Thailand developed to counteract intense tobacco industry interference? What can other LMICs learn from successes in Thailand?

We present a policy analysis showing six core strategies TTCs have used to interfere with Thailand’s tobacco control efforts. These six TTC strategies are: (1) doing business with ‘two faces’, (2) seeking to influence people in high places, (3) ‘buying’ advocates in grassroots organizations, (4) putting up a deceptive front, (5) intimidation, and (6) undermining controls on tobacco advertising, promotion and sponsorship (TAPS) [11]. We describe three examples of TTC interference that Thailand’s public health community successfully counteracted. From this analysis, we identify five effective counter strategies that Thailand’s public health community has used over more than two decades to counteract TTC interference. These strategies include: (1) conducting vigilant surveillance, (2) excluding tobacco companies in policymaking, (3) restricting tobacco companies’ products, sales and use, (4) sustaining pressure on TTCs, and (5) dedicating resources to enforce regulations effectively.

## 2. Background

### 2.1. Brief History of Counteracting TTCs in Thailand

In Thailand, TTCs have been busy interfering in policymaking for many years just as they have in other LMICs. The tobacco control arena in Thailand has been influenced for over two decades by two groups of players. On one side stand the aggressive TTCs and their surrogates along with the somewhat docile state-owned Thailand Tobacco Monopoly (TTM). On the other side stand the governmental and non-governmental health organizations supported by some academics. To understand TTC interference and Thai public health efforts to counteract interference, it is necessary to understand these players and the history of their competition.

From 1939 to 1991, the Thailand Tobacco Monopoly (TTM) was the only legal seller of manufactured tobacco products in Thailand. TTM produces about 20 brands of cigarettes and determines quotas for tobacco growing. In 1989, the American tobacco industry, in its first major attempt to interfere with policymaking in Thailand, worked relentlessly to force the Thai government to open its market to American tobacco products. The industry’s trade arm, the Cigarette Exporters Association (CEA), filed a complaint with the U.S. Trade Representative (USTR) alleging that the Thai government unfairly restricted foreign tobacco companies from selling their products in Thailand. The USTR used heavy-handed tactics to negotiate with Thai representatives on behalf of the CEA, and ultimately pursued the CEA’s case alleging Thailand’s trade protectionism at the General Agreement on Tariffs and Trade (GATT). A small group of Thai tobacco control activists who had been working at the margins of health policymaking became galvanized by this TTC interference and they started to form a wider tobacco control community. In 1991, GATT authorities dealt Thailand a major blow in their ruling that Thailand’s policy was in violation of GATT rules on free trade, despite GATT provisions that allowed governments to protect their public’s health. To avoid U.S. trade sanctions, Thailand decided to open its tobacco market to TTCs [12].

In 1991, Philip Morris (PM), British American Tobacco (BAT), and Japan Tobacco (JT) began distributing their products legally in Thailand. By 2006, PM had increased its market share of sales from about 0% in 1990 to 20.8%, while BAT increased to 2.0%, and JT to 0.4% [13]. TTM’s market share of domestic cigarette sales dropped from 99.4% in 1990 to 75.4% in 2007 [14].

Early on in the trade dispute, Thai tobacco control advocates began to study the American tobacco industry’s interference strategies and tactics by looking at what TTCs had been doing in Central and South America, and by learning from foreign social scientists who had become concerned about the growing consequences of tobacco use worldwide [15,16]. 

At this early stage of efforts in Thailand to counteract TTC interference, it was far from clear how Thailand’s position of self-determination based on prioritizing public health over commercial interests, and protection of national interests, would develop into tobacco control policy. Nevertheless, public resentment of TTC interference gave Thai tobacco control activists leverage to push the Thai government to enact legislation quickly to counteract TTC interference. The Thai government passed two comprehensive tobacco control laws in 1992 based on legislation developed in Canada. In 1993, the government enacted a “tax for health” policy that substantially and prospectively raised excise taxes on manufactured cigarettes to increase their cost. Within two years, Thailand’s new legislation regulated and controlled TTCs more strictly than TTCs had experienced anywhere else in the world. In internal documents, Philip Morris noted that Thailand’s requirements for ingredient disclosure in 1992 were “the first in the world—predating…more recent regulatory developments” [17].

Early on, Thai tobacco control activists were successful in ensuring that the Thai government adopted a policy of excluding tobacco companies and their allies from tobacco control policymaking. However, some TTCs realized that only a few tobacco control activists understood the high-stakes chess match: “… you can see that no one really has an accurate picture of what is happening. Indeed, of all the government officials, National Assembly Members, media, and people in the know in Bangkok, there are only two small groups who understand the game that is being played; a small group of anti-smoking activists who are determined to take down the foreign manufacturers, and willing to take down the TTM (Thailand Tobacco Monopoly) in the process and, the very small group of foreign manufacturers. No one else knows, understands or cares.” [18].

For more than two decades, Thailand’s public health community has vigorously pursued policies to control tobacco use and limit the influence of TTCs (see Table 1). Early measures to counteract tobacco industry interference took place prior to 1995–98 when the Master Settlement in the U.S. produced millions of internal transnational tobacco industry documents that exposed the industry’s practices of interference [19]. In subsequent years, members of Thailand’s tobacco control community invested time and effort to examine tobacco industry policies, both through collaborating with tobacco control advisors outside of Thailand and by looking by themselves at industry documents specific to Thailand. Even before 2003 when researchers in the US had mapped out specific tobacco industry interference strategies [20], Thai tobacco control activists had already showed from documents how TTCs had used both legal and illegal means to manipulate tobacco-related public perceptions and policymaking. Thai tobacco control activists shared this information amongst themselves. Later, researchers conducted more thorough investigations of tobacco industry interference in tobacco industry documents about Thailand [21,22].

**Table 1:** Chronology of Thailand’s Efforts to Stop Transnational Tobacco Company Interference.

Dates	Factors Driving Policymaking	Policies	Accomplishments
1985–1989	Government recognition that tobacco control is a high priority public health issue.	Exclusion of all tobacco industry representatives from policymaking.	FCTC Article 5.3; broader understanding and advocacy in tobacco policymaking.
1990	Public health community’s recognition that TTCs’ interests compete and conflict with the public health mission.	National Committee for the Control of Tobacco use maps of interests.	FCTC Article 5; national coordinating mechanism.
1992	Recognition that TTCs cannot be trusted to voluntarily limit their interactions with government agencies.	Legislation regulating TTC interactions with government agencies.	Laws and regulations on tobacco products and protection of non-smokers. National framework for tobacco control.
1993	Recognition that fiscal resources are necessary to implement comprehensive tobacco control and reduce tobacco use.	Tobacco tax for health.	FCTC Article 6; demand reduction through taxes.
1996	Recognition that TTCs should disclose cigarette ingredients.	Failed attempt to implement ingredient disclosure law, delayed by 5 years of negotiations with TTCs.	-
2000	Recognition that systems management is important.	Thailand Health Promotion Foundation.	Improve effectiveness in implementation.
2001	Recognition that substantial human and financial resources are needed to build comprehensive tobacco control.	Legislation establishing the Thailand Health Promotion Fund through a 2% tobacco and alcohol surcharge.	FCTC Article 26-sustainable funds and advocacy.
2002	Recognition of the need to strengthen regulations.	Improved smokefree policy. New pack warnings proposed.	Extend existing laws.
2003	Recognition of the importance of international commitment.	Thai government ratifies FCTC.	Government and NGO commitment to FCTC.
2004	Recognition that TTCs continue to interfere in government policymaking.	Cabinet directive barring TTCs from making financial or material contributions to government officials, or engaging in political activities.	FCTC Article 5.3; regulation of TTC political interference.
2005	Recognition of a need to close advertising loopholes.	Point-of-sale ban fully implemented.	FCTC Article 13; stronger enforcement.
2008	Recognition of the need to strengthen cessation efforts.	Policy to allocate substantial funding for a national telephone quitline.	FCTC Article 14; smoking cessation support.
2009	Recognition that cigarettes are still not sufficiently expensive to dissuade some smokers from smoking.	Legislation increasing tax on cigarettes.	FCTC Article 6; demand reduction through taxes.
2010	Recognition of the need to coordinate organizations, experts and researchers.	National Strategic Plan on Tobacco Control.	FCTC Articles 21 and 22, Articles 5.3 and 6.

Since Thailand’s public health community began implementing tobacco control measures in 1991, the smoking prevalence rate for men has dropped steadily from 59.3% to 45.6% in 2009, with the average number of cigarettes men smoke declining from 12 sticks per day to 10. Reported female smoking prevalence has remained in the range of 3%, although there is a disturbing upward trend among younger women [23].

### 2.2. Pioneering Tobacco Control NGOs

Action on Smoking and Health Foundation (ASH Thailand) was established in 1986, originally as the Thai Anti-Smoking Campaign Project, the first tobacco control NGO in Thailand. Initially, ASH Thailand’s efforts focused on coping with the US tobacco trade dispute, arguing for robust public health protection in negotiations. When Thailand received the GATT decision favoring the TTCs, ASH Thailand became a driving force in the legislative process that resulted in the Tobacco Products Control Act and the Non-Smokers’ Health Protection Act [24]. For over two decades, ASH Thailand has implemented community-based tobacco control projects throughout Thailand ranging from TTC surveillance to promoting smokefree homes. ASH Thailand works with celebrities, film stars, singers, song writers and athletes to popularize their non-smoking campaign through media and social marketing [25]. ASH Thailand catalogues tobacco news and publishes a monthly magazine to inform the public about TTC strategies and ASH Thailand’s counter strategies.

Thailand Health Promotion Institute (THPI) was established in 1995 as a watchdog NGO under the National Health Foundation. THPI monitors TTC compliance with the law and they inform authorities about violations [24]. THPI regularly conducts studies on tobacco control policy and they disseminate information to the public, notably through booklets documenting unethical TTC strategies and tactics [26]. For example, they alerted government officials about the hazards of a proposal favored by TTCs to privatize TTM. THPI also conducts media advocacy through press releases, letters to the editor, and opinion editorials. In undertaking action, THPI has organized many projects including a youth rally against a PM-sponsored ASEAN Arts Exhibition, and a protest against BAT’s Canal Clean-up Project. 

### 2.3. The Thai Health Promotion Foundation (ThaiHealth)

ThaiHealth was established in 2001 as a result of over six years of planning and advocacy by tobacco control activists and policymakers. ThaiHealth is an outgrowth of Thailand’s early emphasis on policies to reduce tobacco use and to restrict the influence of TTCs. Legislation established ThaiHealth’s mandate and funding through a special surcharge of 2% of excise taxes charged to tobacco and alcohol producers and importers. This revenue, now about US$100 million per year, funds a wide range of health promotion projects, with tobacco control projects receiving about 10% of funds. ThaiHealth plays a significant role in restricting TTC interference because all grantees and partner organizations must pledge not to accept funding from TTCs or their agents. For example, over the past ten years ThaiHealth has increased its sponsorship of sports activities, now with over 200 partners. This policy has effectively shut out TTC sponsorship in venues where they once projected their image and promoted their products freely [27].

## 3. Methods

We conducted this policy study by examining data from three groups with a stake in tobacco control policymaking: (1) tobacco control advocates in Thailand, (2) TTCs, and (3) international tobacco control experts. We gathered information from tobacco control advocates in Thailand in the forms of historical materials (published and unpublished) that the advocates had collected for more than 20 years. We also used secondary reports on the historical development of tobacco control in Thailand. We analyzed documentation of case studies and interviews from assessments of the Thai Health Promotion Foundation in 2006 and from a WHO assessment of national tobacco control policy conducted in 2009 [27,28,29]. We obtained information from tobacco control experts and advisors from published research and past private communication with advisors.

We formulated our analysis of the TTCs’ views about tobacco control in Thailand based on our review of over 300 internal tobacco industry documents that dealt directly with TTC policies. We accessed tobacco industry documents from two online databases (the Legacy Tobacco Documents Library collection and tobaccodocuments.org). We examined these documents from the period 2002 to 2011 and searched the databases using keywords including “Thailand”, “Asia”, “scientists”, “parliament member”, “public health”, “environmental tobacco smoke”, and “regulatory affairs.” Initially, we searched document sources in 2002–2003. Most of these documents have been re-accessed so that current, updated indexing is now cited. We conducted periodic searches in the Legacy collection for subsequent years as new documents were added to the database. Once we located key phrases, persons and institutions, we performed in depth searches following those leads. This procedure resulted in a review of over 3,000 documents from at least 100 individual searches and over 500 pages of printed findings. We obtained interview data in Thailand from interviews we conducted and from findings we reviewed from interviews conducted by tobacco control organizations. Additionally, we analyzed publically available TTC documents (e.g., annual reports) and websites [30]. 

For our analysis, we sought to identify general interference strategies and tactics the TTCs employ over a sustained period. We also sought to identify specific examples of single interference actions TTCs undertook that Thailand’s public health community was able to counteract. We examine how the public health community has pursed regulatory initiatives such as the passage of tobacco control and health promotion legislation, and impeded TTCs from achieving the privatization of TTM or the dismantling of ThaiHealth. We look at the importance of passing strong legislation, achieving implementation and ensuring enforcement when resources can be marshaled, whether to build on an accomplishment or after a setback. We also look at how efforts to pass laws and implement regulations can be delayed by TTC interference. By identifying TTCs’ general strategies and tactics and studying cases where Thailand’s public health community has limited TTC actions, we developed an analysis showing how the public health community has counteracted TTC interference effectively in line with Article 5.3 of FCTC [30]. We look at why it is possible to achieve incremental change to prevent TTC interference. Finally, we examine how tobacco control advocates worked from a position of strength to transform their individual power of agency into structural power.

## 4. Findings

Our analysis shows that in Thailand TTCs have employed six major strategies of interference in health policymaking and tobacco control efforts. We found that TTCs often overlap these strategies to form a complex web of subterfuge. We describe these six strategies below. We also present three examples of TTC interference actions, and we describe how Thailand’s public health community has counteracted such strategies. 

### 4.1. TTC Strategies

#### 4.1.1. Doing Business with ‘Two Faces’

In Thailand, as in many countries, most TTCs pursue their business interests through interference in the political arena. Evidence shows that in Thailand some TTCs do business with ‘two faces’ to fit political and legal circumstances. TTC officials present one face to the public and another face to one another. Often, they say one thing while simultaneously doing the opposite. For example, tobacco industry documents show that TTCs operated in Thailand knowing that they would have to appear to be obeying Thai law prohibiting them from participating in the development of tobacco control legislation. In these documents, they stated that they had influenced the political process through means that were “difficult” and “non-public”, euphemisms for saying that they had conducted illegal hidden lobbying [31]. Another example of the TTC strategy of being two-faced is when the Thai government proposed restrictions on advertising and promotion of TTC products. TTCs wrote a public letter stating their objection claiming that the new law would eliminate their ability to advertise. Yet, when the law was passed, they largely ignored the restrictions and continued advertising and promoting their products as usual, especially at the retail level where they were rapidly expanding their distribution and promotion efforts [32]. The public health community has fought many such battles where TTCs have made public claims about the harm they will suffer contrary to their actual actions, including illegally handing out promotional items, selling clothing with tobacco product logos, installing vending machines that are banned, and distributing window displays of cartons of cigarettes. Policies that set standards against TTC sponsorship of sports in Thailand were valuable in designating areas off limits to TTC promotions. Even when TTCs said they would comply with these restrictions, they used TV broadcasts from outside Thailand for sports sponsorship, a loophole in the legislation.

TTCs also shift their public face on issues in whatever direction they feel will gain them the most favorable press while advancing strategies they hide from the public. Our research of the industry documents shows that in Thailand TTCs had taken serial conflicting public positions about their products, namely, that the health dangers of smoking are not clear; everyone knows the health dangers of smoking; tobacco is not addictive; tobacco is addictive but addiction is not important; smuggling tobacco products is their legitimate right to provide their quality product to the public; they do not condone or foster smuggling and regularly help governments to stop it [33,34].

Increasingly, as the Thai public and government demand that corporations behave according to higher ethical standards, TTCs have adopted policies and practices that they say are evidence of their sense of corporate social responsibility (CSR). For example, PM has stated, “Today, the Philip Morris family of companies is one of the leading supporters of environmental protection and conservation… ranging from 12 countries in Central and South America to projects in…Thailand and Malaysia” [35]. PM is not alone in supporting environmental causes in Thailand. The TTM also supports many environmental and educational programs in schools throughout Thailand. TTCs present their CSR policies and practices as part of their public face, seeking to divert the public’s attention away from the detrimental effects of their products while never conceding that they have any responsibility for the harm their products cause [36]. The TTCs’ public face of ‘CSR’ policies and practices is contrasted by their private face of moral disengagement and relentless pursuit of legal maneuvers to protect themselves from product liability actions or punitive legal decisions [37]. This two-faced business practice is one reason that the Thai public and the business community in Thailand see TTCs as among the most disreputable and unethical of companies [38].

#### 4.1.2. Seeking to Influence People in High Places

TTCs have a long track record of trying to establish a positive regulatory climate for their operations. In Thailand, there are documented cases where TTCs have supported academics and politicians, as well as TTC ‘enemies lists’ of anti-smoking activists [39]. The most obvious kind of TTC interference is when TTCs enlist influential persons to side with them in supporting or opposing an action. Numerous Thai politicians have been influenced by TTCs, particularly those who have constituents with a stake in tobacco production in Northern Thailand. Other politicians have chosen to passively do the bidding of TTCs by delaying tobacco control legislation under the weight of TTC pressure or support. Some TTCs have tried to influence each Minister of Public Health and other high-level officials in key positions. They have attempted, sometimes successfully, to meet privately with Ministers of Public Health to gain their cooperation in violation of the spirit of Thai law that restricts TTCs from participating in policymaking. In one internal tobacco industry document, a TTC representative reported on meeting with the Thai Minister of Public Health saying, “I believe the present Minister offers us the opportunity to contribute our views and our success will depend on the degree to which we can educate him and his department on these issues, without overburdening him with details in which case he would probably seek clarification from our opponents” [40]. In a similar effort, TTCs have hired ‘independent’ experts and academics to persuade Thai officials that secondhand smoke (SHS) is not dangerous [41]. There is a long history in which academics in Asia have also been co-opted into accepting industry ‘expert’ opinions about secondhand smoke. TTCs have enlisted some Thai scientists in this effort, and through commission or omission other scientists have allowed themselves to be used by TCCs for their purposes [42]. 

By seeking to influence people in high places, TTCs have been able to delay and in some cases entirely derail major tobacco control initiatives in Thailand [43]. TTCs operate under the assumption that many high-level government officials tend to blindly follow WHO recommendations with little understanding of what is at stake [44]. Thus, TTCs try to convince officials that their understanding of tobacco issues is superior to that of WHO staff. TTCs have been effective at persuading some Thai politicians and academics to take actions to undermine tobacco control. TTCs have also successfully enlisted some politicians and academics to advocate openly for TTC interests, although most politicians and academics shy away from blatant advocacy because they fear it will damage their reputation [45]. Some TTCs have been successful at simply getting officials to accept bribes, as in the example we describe below. All of these tactics constitute interference in policymaking. They stem from a pervasive view in the corporate world: Corporations do not need to be accountable to anyone except stockholders because the ethics of conduct for private firms are generally less demanding than those applied to public agencies. This is a false argument that TTCs repeatedly cite to defend their actions. 

#### 4.1.3. ‘Buying’ Advocates in Grassroots Organizations

TTCs have long tried to create a perception in the public eye that they contribute responsibly to society. In Thailand, they pursue this goal by transferring some of their profits to their philanthropic foundations and then publicizing heavily their strategic philanthropic activities. Recently, TTCs have given financial support to social causes that many people believe are worthwhile (e.g., environmentalism, human rights). In Thailand and throughout Southeast Asia, PM has emphasized its support of social causes and portrayed itself as a “community of caring people” [28]. In some cases, TTCs have funded small community projects. In other cases, they have supported highly recognized NGOs like the Population and Community Development Association, headed by famous AIDS activist Mechai Viravaidya, that is committed to reducing poverty in Thailand [46]. TTCs employ this strategy of ‘buying’ advocates in grassroots organizations because they can exploit groups that are desperate for resources. Their strategic philanthropy capitalizes on a common misconception that those who support good work must be good themselves. By associating themselves with worthy causes, TTCs attempt to create a perception that they are honest, caring organizations. Tobacco industry documents show that TTCs routinely strive to create such associations. TTCs claim there are no strings attached to the support they offer, but they hide their public relations motives from those they support and from the public [47]. In actuality, TTCs often expect a quid pro quo: That the groups they have supported will speak out on their behalf by offering testimony about their beneficial actions for society [48]. The public health community in Thailand has found an effective way to counter this strategy, beyond simply exposing it. ThaiHealth supports a wide range of social development projects, including ones that seek to improve the environment and social well-being. ThaiHealth requires, as a condition of support, that grantee and partner organizations that receive ThaiHealth funding not accept support from TTCs or their surrogates [27]. This has greatly reduced TTCs’ ability to employ this strategy. 

#### 4.1.4. Putting up a Deceptive Front

TTCs use front groups (fake grassroots organizations) and surrogates to interfere with policymaking. Some TTCs in Thailand employ this patently unethical public relations practice to further their corporate interests. For example, in the 1990s, Gray Robertson of Healthy Buildings International (of Australia) went around Asia, sponsored by PM, to talk about ‘sick building syndrome’ with the undisclosed objective of trying, on behalf of PM, to dissuade officials from giving attention to indoor SHS exposure. Robertson was promoting himself as an “independent expert” and he was aided in this deception by two academics working for PM who lectured on air quality issues in Thailand and other Asian countries. In actuality, his purportedly ‘independent’ air quality/ventilation business was mostly a front for TCCs who were pursuing their objective of delaying or stopping SHS regulations. In Thailand, most of Robertson/PM’s work using this strategy came after the 1992 Nonsmokers’ Health Protection Act had already been enacted, so legislation already mandated the reduction of exposure to SHS. However, subsequently “independent experts” were successful in causing a delay in updating SHS regulations because they convinced Thai officials and academics to give limited attention to monitoring and enforcement of the ban on indoor SHS pollution [41].

#### 4.1.5. Intimidation

TTCs use intimidation not simply to exhibit their might, but often to destroy the reputation and career of anyone they feel threatens them. Tobacco control experts and advisors have highlighted how TTCs relentlessly intimidate politicians, academics, and public and private organizations to coerce them into following their policy agenda at the risk of facing harsh consequences [49]. Tobacco industry internal documents show that TTCs will make every effort to see that politicians who oppose them are defeated. They do this to destroy their perceived enemies and to intimidate other politicians [50]. In Thailand, TTCs intimidate officials by threatening to file law suits and legal challenges. Their strategy has been effective. For example, when members of parliament were crafting provisions of the Tobacco Product Control Act, TTCs used intimidation to see to it that the product disclosure provision was not supported. When the Act was eventually passed, the product disclosure provision of the legislation was gutted. In another case, TTCs gained powerful international support from the EU, the US, and the UK to pressure the Thai government and tobacco control advocates to extract assurances from the Thai government that officials would not release lists of cigarette ingredients to the public, even though the TTCs had supplied this information to the government [22]. 

#### 4.1.6. Undermining Controls on Tobacco Advertising, Promotion and Sponsorship (TAPS)

TTCs oppose restrictions and bans on TAPS because they see their very existence tied to TAPS. For example, a PM report about public relations strategies stated:

If one takes the pessimistic view of present trends, the tobacco industry could lose all its political clout within two years. Overstated? Not really. If you take away all advertising and sponsorship, you lose most, if not all, of your media and political allies. If you take away those freedoms, there is hardly any barrier to a punitive tax regime, part of it going to fund our complete anathematization through the funding of ever more extreme anti-campaigns courtesy of ‘social levy’ foundations, of which there is a growing fashion [51].

In Thailand, where bans on TAPS were instituted nearly two decades ago, TTCs continue to undermine any law or regulation banning TAPS. In 1992, TTCs claimed that if the Tobacco Product Control Act were passed, advertising as they commonly practiced it would be prohibited. Yet after the Act was passed, many TTCs continued their business-as-usual promotion of their products, especially at the retail level. It was not until 2005 when the Thai government began enforcing the ban on tobacco retail displays that the TTCs began begrudgingly complying [52]. Even today, TTCs continue to promote their products illegally at some sports, music and entertainment venues. Their “cigarette girls” still work at nightspots, and TTCs still promote their products to individuals through the Internet [53]. PM sponsored the ASEAN Art Awards, and later mentioned in internal documents that they were able to influence the ASEAN secretariat on trade issues because they had invested in this program [54].

Clearly, TTCs continue to violate TAPS laws because they desperately need to reach new customers [47]. It is of equal concern that they violate these laws to try to maintain a public perception that their products are harmless and desirable. This insidious practice is designed to garner public support to legitimize their practice of interfering with policymaking. 

### 4.2. Case Examples

Three examples of tobacco company interference in Thailand include: (1) a large donation a TTC made to the Ministry of Education, (2) a tobacco industry conference and trade show called “TabInfo Asia 2009” held in Bangkok, and (3) bribes a TTC paid to Thai officials responsible for purchasing tobacco for the Thai Tobacco Monopoly.

#### 4.2.1. A Donation to the Ministry of Education

In 2003, PM made a 3,000,000 baht (about 72,000 US dollar) donation to the Ministry of Education (MoE) for school programs. PM publicized their philanthropic gesture widely. The donation was in fact an attempt to influence people in high places and an effort to promote their company image in the policymaking arena. The Thai public health community was effective in detecting and counteracting this strategy. ASH Thailand lobbied the MoE intensely until the MoE decided to return the donation to PM [55]. This counter action was successful because ThaiHealth has officials from many government ministries, including the MoE, who served on its board. Government agencies that receive grants from ThaiHealth are required to commit to not accepting support from TTCs. This provision makes government agencies accountable and reduces TTC attempts to co-opt individuals and institutions [26]. However, even though TTCs were well aware of this provision, they still brazenly attempted to make the donation, and the MoE initially accepted it.

#### 4.2.2. TabInfo Asia 2009

In 2009, Thai tobacco control groups learned that the tobacco industry was planning to hold a conference and trade show in Bangkok. The industry uses such events strategically to boost their image in a country or region, influence people in high places, promote their products indirectly to a wide audience, and show that they are an intimidating business force that can operate without the acceptance of the government or NGOs. The industry expected TabInfo to draw up to 3,000 participants.

Several Thai governmental agencies and NGOs opposed the event and countered the industry’s strategies. The government declared that TabInfo was a public event, therefore making Thailand’s laws prohibiting tobacco advertising and product display enforceable inside TabInfo. NGOs organized several levels of protest. Youth demonstrated at the event site with tobacco control activists from neighboring countries. NGOs gained extensive local and international media coverage. At least 300 articles appeared in Thai print news as well as radio and television coverage, made possible by strong contacts Thai tobacco control NGOs have developed within the mass media over many years.

Less than 900 participants actually attended the event, making it a costly failure for the industry. Thai government officials did not participate in the event. TTM converted their anchor megapavilion from a product showcase into a “Welcome to Thailand” visitors center. Thai police arrested and fined trade show organizers for violating Thai laws, the first time that tobacco exhibitors had ever been held accountable for advertising at their own trade show. The industry’s image was decidedly tarnished. TTCs were unable to influence people in high places, promote their products or intimidate the Thai public health community. In addition, the protest brought together representatives from the Southeast Asia region for training and to establish a surveillance system to control industry interference according to provisions of FCTC Article 5.3 guidelines [56].

#### 4.2.3. Bribing Thai Officials

In 2010, Dimon, Inc. and Standard Commercial Corporation (now merged together as Alliance One, one of the largest international tobacco leaf suppliers) paid more than US$1.8 million in bribes to Thai tobacco officials at TTM to obtain over $18.3 million in sales contracts. The US Securities and Exchange Commission initially reported the bribe. Subsequently, the Thai Department of Special Investigation undertook its own investigation [57]. While it is common in Thailand for businesses to seek to influence people in high positions, this case shows that TTCs brazenly offer politicians and officials big favors to achieve their ends.

These are just three examples that illustrate TTCs’ aggressive, corrupt business practices, and their disregard for tobacco control policy. The examples illustrate how TTCs often employ a strategy that fulfills multiple goals. Importantly, the examples show so ways that tobacco control advocates have thus far been able to thwart TTC efforts to undermine tobacco control. Advocates have successfully derailed TTC efforts to establish a joint venture with TTM, to covertly promote the privatization of TTM, and to attempt to orchestrate the dismantling of ThaiHealth.

Past TTC practices offer important clues about strategies and tactics TTCs may use to interfere with tobacco control in the future. We now turn to a discussion of ways LMICs can prevent, detect and counteract TTC interference. 

## 5. Policy Implications

Our analysis shows that tobacco control advocates will be wise to be highly vigilant about TTC activities because TTCs employ multiple strategies in the sociocultural, commercial and political environments, and they invent new subversive approaches. It is important to be proactive about anticipating TTC strategies while being ready to respond swiftly to counteract their interference. While policymaking and legislation are essential, proactive efforts should go far beyond simply declaring policies and enacting laws that are consistent with the FCTC.

A comprehensive approach is preferable because it deals with TTC efforts to interfere with tobacco control at multiple levels from different angles. A comprehensive approach involves being vigilant while simultaneously undertaking policy innovations that will prevent TTCs from circumventing or undermining FCTC-based policies. It is important to make every effort to exclude TTCs from the policymaking arena so as to prevent them from taking unacceptable actions. It is also essential to conduct a thorough assessment of TTC vulnerabilities to limit and ultimately stop their interference.

About two decades ago, the Thai Government and tobacco control leaders began establishing a comprehensive set of countermeasures that now serve as a template for success. They include tobacco control legislation, ingredient disclosure, tax policy, sustainable funding for tobacco control, cigarette pack warnings, ban of point of sale display, and designation of smokefree public and private places. At the same time, the government established a “firewall” to restrict TTC involvement in tobacco control policy—an aggressive stance for preventing TTC interference. The government and tobacco control leaders continue to develop policies that restrict TTCs activities and limit their ability to implement new strategies. We now describe some recent innovations.

### 5.1. Policy Innovations to Counteract and Prevent TTC Interference

In Thailand, the public health community recently developed a National Strategic Plan for Tobacco Control for 2010–2014 (NSPTC) under the auspices of the National Committee for the Control of Tobacco Use (NCCTU). The NCCTU was established in 1989 to form tobacco policies and implementation guidelines, carry out management and coordination, monitor and follow up on tobacco control measures, enforce tobacco control laws, and appoint subcommittees [58]. NCCTU is chaired by the Ministry of Public Health (MoPH) with representatives from professional organizations, health-related NGOs, related ministries and the media.

One of the eight areas of the National Strategic Plan for Tobacco Control is surveillance and control of TTC interference [59]. Within this area, there are seven strategies, each with one or more activities. The activities follow many of the suggested measures in FCTC Article 5.3 guidelines [3]. The following is a list of the most important activities underway in Thailand.

1. Increasing public awareness of regulations prohibiting TTC interference.
2. Monitoring public relations, government and TTM contacts with the TTCs, and TTC CSR activities.
3. Monitoring vested interest groups that work with TTCs.
4. Issuing notifications of regulations prohibiting importation, manufacturing, or sale of new types of tobacco products.
5. Informing the public of marketing and other threats from TTCs.
6. Establishing surveillance networks of TTC activities down to the community level.
7. Taking legal action on new or “below the line” marketing techniques.
8. Researching, regulating, and campaigning against tobacco industry CSR activities.
9. Monitoring TTC glamorization of tobacco.
10. Educating the public to understand that tobacco use is a non-normative behavior.
11. Monitoring TTC legal violations, taking legal action, and publicizing prosecutions and penalties.

It is important to note that in Thailand prior to 2000, existing tobacco control authorities, ASH Thailand, THPI and other NGOs had undertaken at least five of these actions (i.e*.*, 1, 2, 5, 8, 9). To realize these goals over the next two years, a wider group of organizations and agencies have joined together to produce research and counteract TCC interference.

Following the approval of the NSPTC by the NCCTU in 2010, the MoPH formed 12 NSPTC implementation committees under the coordination of the Bureau of Tobacco Control (BTC) [60]. BTC was established in 1991 in the Department of Disease Control, MoPH (originally as the Tobacco Consumption Control Office) to regulate Thailand’s newly opened tobacco market according to provisions in the Tobacco Product Control Act [24]. Tobacco industry surveillance is one of BTC’s main activities. BTC investigates complaints about potential TTC violations of law with provincial public health offices and 12 regional disease control centers. Individuals can report potentially illegal TTC activities directly to the BTC through a call center or through the Internet. One of the BTC’s most important projects is to develop and operate a National Surveillance System that includes four activities: conducting law enforcement, studying tobacco consumption patterns, monitoring media, and increasing surveillance of TTC. The BTC has designed this system to consolidate information from active and passive monitoring sources, centrally and regionally, and to develop a database for tobacco control research [59]. 

One of the 12 NSPTC implementation committees has a mandate to monitor and control TTC interference in tobacco control policymaking, and to take legal actions when necessary. Membership of this committee consists of 23 officials representing professional institutions, health-related NGO’s, and academics, including from the Excise Department, Council of State, and Ministries of Commerce, Education, Interior, Tourism and Sports, and Culture [59]. In addition, committees other have mandates that contribute to the surveillance of TTCs.

In the NSPTC, many key activities require surveillance and monitoring of TTC activities at the regional, national, and local levels, and assessment of TTC vulnerabilities. These research activities have to be coordinated in a network and supported on a long-term basis. One important policy innovation has been to build a research network focused on studying TTCs and to create sustainable mechanisms for supporting such research.

Thailand-based tobacco control advocates participate in a regional network called the Southeast Asia Tobacco Control Alliance (SEATCA), established as an NGO in 2001 to support comprehensive tobacco control in seven Southeast Asian countries. SEATCA brings together representatives from NGOs, research institutions and governments. SEATCA coordinates and supports research on TTCs to improve tobacco control laws; expand smokefree environments; and restrict TAPS. SEATCA’s strategies focus on: (1) building up local evidence bases; (2) enhancing local capacity building and mentorship; and (3) developing policy through research dissemination. SEATCA has sponsored events to advance FCTC guidelines on limiting TTC interference, including publishing the “Surveillance of Tobacco Industry Activities Toolkit” and “Tobacco Industry Interference in Health Policy in ASEAN Countries” [61,62].

Monitoring TTC activities is often difficult and requires painstaking attention to details. To be effective at identifying subversive TTC interference, it is important to have ongoing credible research that informs policymaking. Since the FCTC guidelines were established, Thailand’s public health community has moved to comply with tobacco control best practices specified in WHO’s MPOWER indicators [63,64]. To this end, the Tobacco Control Research and Knowledge Management Center (TRC) was established in 2005 as an academic research center at Mahidol University’s Faculty of Public Health. Funded by ThaiHealth, TRC promotes and supports research on and surveillance of TTCs among other topics. TRC works to discredit and debunk misinformation the tobacco industry provides.

Recently, SEATCA and TRC jointly launched the Tobacco Industry Surveillance and Study Group (TISSG) to advance previous efforts to analyze TTC policies and actions. One innovative approach to research is that TISSC-sponsored researchers, including young investigators, increasingly collaborate with international researchers on policy evaluation studies such as the present study [65]. TISSC has established Internet-based monitoring and reporting systems to compile up-to-date surveillance data [63]. Using these data, TISSC has mapped TTC interference strategies and tactics in Thailand and regionally [66].

### 5.2. Guidance for LMICs

Our analysis of the history of efforts in Thailand to control TTC interference reveals an inspiring story of possibilities for LMICs. For the first decade of tobacco control in Thailand, a committed group of less than a dozen full-time tobacco control advocates managed to mobilize social support for tobacco control, gather evidence, make specific claims against TTCs, define the principles of engagement, and push unceasingly for ever stronger legislation and enforcement. Since the first confrontation at GATT, they have also sought the advice of international experts to learn about strategies tobacco control advocates have used abroad to counter TTC interference. This small group pursued their goals with a clear-eyed understanding that TTCs would neither accept evidence proving the dangers of their products nor act ethically if they were allowed to participate in tobacco control policymaking. This small group also recognized that TTCs were willing to use any means to pursue their goals, including using legal action, obstruction, harassment, slander, influence buying, bribery, fraud and conspiracy.

Our analysis also shows that tobacco control advocates recognized that there were limits to the amount and type of influence they could have as a small group of individuals. Therefore, in the second decade, they converted some of their individual power of agency into structural power by working to establish a tax-supported health promotion foundation. Tax-supported foundations can provide stable resources for tobacco control. They can also expand the community of partners engaged in exposing TTC interference and holding TTCs accountable. Moreover, such foundations can be a vehicle for integrating tobacco control into the larger national agenda for development. In Thailand, the advent of ThaiHealth has transformed the public health landscape; rather than a few activists working on a single health issue, Thailand now has a wide-range of partners with broader interests conducting tobacco control.

Emerging evidence from studies in LMICs shows that TTCs cannot be trusted to behave as good corporate citizens, follow tobacco control laws or comply with polices [67]. FCTC provides an international framework for national action to keep TTCs in check and hold them accountable, but in many LMICs, TTCs can still manipulate political systems and circumvent or co-opt legal infrastructures. Thus, at a fundamental level it is important for tobacco control advocates to work to strengthen their political and legal systems as much as possible so that stronger tobacco control legislation and regulations can be enacted. Of course, this takes time and effort. 

In most LMICs, TTCs can pursue their interference strategies successfully because of a lack of enforcement of existing laws and policies. Police and other enforcement agencies are often underfunded and uninterested in enforcing tobacco control laws. In some cases, they also participate as a partner with TTCs in corruption. So, tobacco control advocates have to push relentlessly to make it impossible for police and other law enforcement agencies to avoid enforcing laws. In Thailand, tobacco control advocates have worked for many years to improve the basic legal framework so that tobacco control could be more effective. For example, they have made it impossible for government agencies to avoid enforcing the point-of-sale display ban. In the TabInfo Asia 2009 case, they pushed in public, through media coverage, to make it impossible for the police to avoid taking action. These successes have encouraged tobacco control advocates in other LMICs to work on improving their legal systems while pursuing aggressive enforcement actions against TTCs consistent with FCTC provisions.

In LMICs, long-term support from political sponsors is an essential ingredient for reducing TTC interference. Experience in Thailand shows that tobacco control advocates have been successful because they seize political opportunities as they emerge and they generate political support when it is necessary. Advocates have found that politicians with differing political philosophies are sometimes surprisingly willing to support tobacco control legislation that is based on sound principles. Such politicians are especially cooperative if their support offers the prospect of popular recognition and widespread approval from their constituents. This shows that it is important to cultivate ethical relationships with politicians regardless of political affiliation, and to give politicians recognition for their support. This, however, should not require that advocates align themselves too closely with one politician or party, with the implied expectation of reciprocity in other political matters.

In attempting to prevent and counteract TTC interference, it is important to “inoculate” and mobilize the general public against TTC actions through health promotion. In Thailand, everyone from high-level government officials to common citizens is involved in health promotion. One important focus of health promotion in Thailand is providing a steady stream of credible information about TTCs to the mass media. Twenty-five years of tobacco control campaigning has had a remarkable effect. A recent survey shows that a large majority of smokers have a negative view of smoking, and they support smokefree public places, support a complete ban on advertising, and support services to help smokers quit smoking [68].

Counteracting TTC interference depends on a wide and deep commitment at the level of development policy and systems change, not just on the efforts of a few NGOs or the regulatory efforts of a few governmental agencies. As Beaglehole, an expert on health systems change has emphasized, “In the long term, monitoring and accountability are necessary, but not sufficient for implementation of mutually synergistic interventions as shown with the slow implementation of the Framework Convention on Tobacco Control; … action should emphasize integration of … interventions into national development processes and the actions of multilateral institutions” [69].

Stakeholders in LMICs working to integrate tobacco control into the mandates of many agencies may likely face similar TTC interference strategies as those encountered in Thailand. This is because TTCs tend to replicate their strategies across LMICs. To provide LMICs with specific guidance about how to apply our findings to their own efforts to counteract TTC interference, we offer the following recommendations and descriptions of options.

### 5.3. Recommendations and Options for Low- and Middle-Income Countries

1. *Establish legal mechanisms to regulate and monitor TTC all interactions with all government agencies. *Since tobacco control is a public health issue, legal mechanisms often focus on public health law. Codify in law restrictions on interactions with TTCs that apply to all government agencies and officials. Develop a proactive legal framework that anticipates TTC interference strategies seeking to influence people in high places.
2. *Ensure transparency and accountability in government.* Working transparently with partners inside and outside of government is essential to maintaining a united front for prohibiting TTC efforts to influence people in high places. It is important to establish specific policies limiting TTC interactions with government agencies through publically disseminated rules of engagement for all government and public-supported agencies, including the rejection of TTC-sponsored CSR projects.
3. *Seek robust, transparent policymaking to avoid TTC stalling tactics and efforts to dilute legislation. *Opportunities to take action against TTCs are often time-sensitive, so it is important to act quickly when favorable political conditions for strong action are present. Establish an advocacy plan and timetable that does not give TTCs opportunities to use intimidation to derail strong legislation and regulation.
4. *Establish sustainable funding for research and surveillance. *It is important to identify, study and expose TTC interference. Sustained research and surveillance are cost effective for society. Earmarked taxes on all types of tobacco products equivalent to at least 75% of the retail price are an effective way to support tobacco control research, guarantee resources for tobacco control projects, and build tools to minimize TTC political interference.
5. *Squelch influence buying in academia and civil society. *Work to gain cooperation from partners to follow rules limiting interactions with TTCs to reduce their influence on important people, reduce their ability to buy advocates in grassroots organizations, and put up a deceptive front. Establish consequences for groups and individuals who accept money or other support from TTCs, or who work as surrogates for TTCs.
6. *Build public support for tobacco control.* When official enforcement is weak, an effective alternative can be to engage the general public in monitoring of TTC non-compliance with laws and policies. Build a cadre of watchdogs in the public and constantly highlight the benefits of tobacco control in the media while exposing TCCs’ subversive practices in public.
7. *Promote innovation and broad implementation through strategic partnering.* Tobacco control measures often result in important societal benefits in addition to the improvement of the public’s health. Tobacco control advocates should seek to identify a wide range of stakeholders beyond the field of tobacco control who will be willing to integrate tobacco control into their projects from the national level to the community level. It is important to enlist experts and authorities who support innovative tobacco control measures and who will champion the benefits of confronting TTC interference.
8. *Denormalize tobacco use to counteract TTC image-making. *Counteract TTCs by working on actions that reduce the overall acceptance of tobacco and diminish the credibility of TTCs. Work through these actions to counteract TTC attempts to undermine controls on TAPS. Such actions should include establishing 100% smokefree places, requiring large picture pack warnings, and prohibiting store displays of cigarettes. Use research and advocacy to educate policymakers so that denormalization measures are adopted.
9. *Upgrade laws to meet all WHO FCTC requirements and guidelines.* Understand the political process and map out a political strategy to establish laws for implementing FCTC measures. Building legal support for FCTC provisions may require informing and lobbying policymakers over a long period of time. Work on multiple fronts to show policymakers your determination to move tobacco control through the legislative process. Seek out political supporters regardless of their political affiliation.
10. *Monitor TTC activities.* Build a coalition of marketing, economic, legal and public health experts to monitor TCCs and take preemptive action against TCC interference. Identify front groups and individuals who are likely to be complicit in TTC practices of doing business with two faces by misrepresenting the consequences of tobacco control measures or acting subversively to allow TTCs to put up a deceptive front.
11. *Foster cooperative efforts among interested parties while excluding TTCs.* TTCs often try to pit tobacco control advocates and organizations against each other on important policy issues. Building cooperation therefore comes from being aware of how TTCs seek to manipulate tobacco control partners and interested parties in policymaking. Constantly educate partners and interested parties by providing current examples of TTC interference, and highlight successful strategies for counteracting interference.

## 6. Conclusions

FCTC has raised tobacco control advocates’ awareness about how TTCs attempt, often successfully, to interfere in policymaking. But awareness is not enough. Even now, in an era when TTCs are confronted with the first international tobacco control treaty endorsed by over 170 countries, they continue to subvert, delay and destroy policymaking efforts to reduce tobacco use. TTCs are especially aggressive in their attempts to interfere in policymaking in LMICs where the potential for profits is the highest, and where political and legal structures are typically less than robust [70]. Experience in Thailand shows that TTCs employ multiple strategies to ward off any restriction of their business activities, and they exhibit persistent ingenuity in their efforts to avoid following laws or complying with policies.

The public health community in Thailand, coming together from governmental and non-governmental agencies, has made important gains in preventing and controlling TTC interference. Today, Thailand is already in compliance with many provisions of FCTC and the MPOWER indicators [71]. The gains have been hard won. Thailand’s compliance with FCTC is the result of more than two decades of sustained, dogged effort.

Since TTCs are multinational corporations with the habit of testing and replicating their strategies around the world, LMICs can anticipate that TTCs may be using the same interference strategies that we have identified in Thailand. Experience in Thailand shows that it is possible for tobacco control advocates, through careful study and persistent effort, to establish structures and systems for preventing and counteracting TTC interference. Just as TTCs employ strategies according to country-level and local conditions, so too is it necessary for national public health communities to critically assess what policies and measures make sense in their context. FCTC provides guideposts. However, basic compliance with FCTC should not be the goal. Only creative thinking, greater ingenuity and vigilance will take us beyond FCTC’s protocols and guidelines to the point where TTCs find it counter-productive to interfere with policymaking. Instituting measures to stop TTC interference is a goal worth aiming to achieve, just as nearly two decades ago two legal experts aimed for the goal of persuaded people around the world that an international treaty on tobacco control was worth achieving [72].
